# Pharyngeal-cervical-brachial variant of Guillain-Barré syndrome: a case report of a rare complication following Dengue-Chikungunya co-infection

**DOI:** 10.11604/pamj.2021.38.356.28363

**Published:** 2021-04-14

**Authors:** Osama Mohiuddin, Anosh Aslam Khan, Syed Hamza Bin Waqar, Ali Tariq Shaikh, Momina Mariam Marufi, Sumeen Jalees, Farah Yasmin

**Affiliations:** 1 Department of Internal Medicine, Dow University of Health Sciences, Karachi, Pakistan,; 2 Department of Internal Medicine, State University of New York, Down State Medical Center, Brooklyn, New York, USA

**Keywords:** Guillain-Barré syndrome, Dengue, Chikungunya, co-infection, case report

## Abstract

Pharyngeal-cervical-brachial (PCB) variant of Guillain-Barré Syndrome (GBS) is characterized by weakness in cervicobrachial and oropharyngeal region, together with areflexia of upper limbs. Being an uncommon variant, it is often misdiagnosed as other neurological conditions resembling GBS. Although most of the cases occur as a post-infectious complication, no reports describing its development following dengue-chikungunya co-infection have been documented. A young female presented with a progressive history of swallowing difficulty, bilateral arm weakness and neck weakness. Three weeks earlier, she was presented with clinical features corresponding to dengue and was symptomatically treated. Currently, hypotonia and decreased muscle strength were observed in both upper limbs and neck. Detailed investigation revealed the presence of Immunoglobulin M (IgM) antibodies against dengue antigen (NS 1) and Chikungunya virus (CHIKV), confirming the possibility of previous dengue-chikungunya co-infection. Nerve conduction studies and electromyography of upper limbs pointed towards findings consistent with the early stages of acute motor demyelinating and possible axonal neuropathy. The detection of antiganglioside antibodies (anti-GT1a antibodies), confirmed the diagnosis of the pharyngeal-cervical-brachial variant of GBS. A five days treatment of intravenous immunoglobulin (IVIG) along with physical rehabilitation was started which led to significant improvement and the patient was discharged after 15 days. PCB is an unfamiliar variant of GBS for many clinicians. Diagnosis can be made by a thorough history, clinical examination and investigations that can rule out other potential causes of cervicobrachial and oropharyngeal weakness. It also necessitates careful monitoring and followups after mono- and co-arboviral infections to prevent any debilitating neurological complications.

## Introduction

Pharyngeal-cervical-brachial (PCB) variant of Guillain-Barré Syndrome (GBS) is characterized by weakness in cervicobrachial and oropharyngeal region, together with areflexia of upper limbs. Pure PCB was first described by Ropper in 1986, after studying three patients with progressive weakness of oropharynx, neck and shoulder, with the absence of sensory loss and lower limb weakness [1,2]. As GBS often present itself as an array of acute inflammatory polyradiculoneuropathy, motor weakness and diminished reflexes, PCB being an uncommon variant, sometimes escapes diagnosis or often misdiagnosed as myasthenia gravis, botulism and other conditions mimicking the general presentation of GBS [2,3]. Apart from “pure PCB”, some patients with PCB-like symptoms might have overlapping clinical features of Fisher syndrome (FS) and Bickerstaff brainstem encephalitis (BBE). These patients have additional symptoms of limited eye movement, generalized areflexia and severe ataxia. In the cases of PCB overlapping FS, consciousness is intact while it remains altered in cases of PCB overlapping BBE. This shows that PCB can form a continuous spectrum with FS, BBE and GBS, and is mostly considered as a diagnosis of exclusion [4].

Studies have shown that 70% of the PCB cases follow upper respiratory infections along with serological evidence of Campylobacter jejuni infections in 31% of the patients. Other less commonly preceding infectious pathogens include mycoplasma pneumonia, cytomegalovirus, Epstein-Barr virus, Haemophilus influenza [5]. Furthermore, recent studies have also described the occurrence of PCB following dengue and chikungunya infection [6,7]. The first case of dengue and chikungunya co-infection was reported in Thailand in 1962. Over the years, research showed that both the viruses are transmitted by Aedes species of mosquitoes, which is a major reason for their overlapping geographical and temporal epidemiological profile, mostly in Asian and African regions. Moreover, the dengue-chikungunya co-infection often result in acute febrile syndrome with non-specific features like arthralgia, myalgia and fever. However, neurological disorders in cases of co-infection are uncommon [8]. Herein, we describe the first case of the pharyngeal-cervical-brachial variant of GBS, occurring as a complication secondary to dengue-chikungunya co-infection.

## Patient and observation

A 31-year-old female presented to the emergency department with a 4-day history of swallowing difficulty and a 3-day history of bilateral arm weakness and neck weakness. Initially, she had swallowing difficulty with only solids which progressed to liquids by the end of the third day. It was also associated with drooling of saliva, dry cough, nasal speech and nasal discharge. Her swallowing difficulty was continuous. There was no complaint of shortness of breath. She also had neck weakness and difficulty in head rotation. The weakness in arms was progressive in intensity, continuous and painless. She denied any associated symptoms of fever, sore throat, double vision, sensory facial disturbance, hearing difficulty, gait disturbance, diarrhea and vomiting. There was no significant history of dietary change, travel or medication use. Bowel and bladder functions were intact. There was no family history indicative of a similar pattern of symptoms with the complaint of fever, headache, generalized body ache, vomiting and fatigue for four days. She had a non-significant history of chronic illness except three weeks earlier, when she presented with complaints of persistent high-grade fever, throbbing headache, large joints pain and severe generalized fatigue for five days. Based on history and clinical grounds, a differential diagnosis of dengue was made. The patient was admitted and administered intravenous normal saline infusion, analgesics and antipyretics. After three days of management, the fever subsided along with significant improvement in other symptoms.

On examination, the patient was fully alert, oriented and vitally stable with SpO2 of 98%. The extraocular movements were intact in all directions with normal convergence. The pupils were bilaterally equal and responsive to light and accommodation. Her vision was normal as well. There was no nystagmus in any direction. Bell´s phenomenon was observed in the left eye, with loss of left-sided nasolabial folds and forehead wrinkles, indicating towards lower motor neuron type of facial palsy on the left side. Gag reflex was also weak with a right-sided deviation of uvula. No fasciculations were observed on tongue protrusion. The facial sensation was intact. Rest of the cranial nerve examination was unremarkable. On motor examination of upper limbs, the bulk was normal bilaterally. Hypotonia was observed in both upper limbs. Muscle strength was assessed through the Medical Research Council (MRC) scale, which was 2/5, proximally and distally in both limbs. Deep tendon reflex (DTR) was significantly reduced. The power of neck muscles was reduced with a few episodes of head drops. The movements of the neck, however, was completely impaired against resistance. On lower motor examinations, the bulk, tone, power and DTR were normal. The sensory and cerebellar examinations were also unremarkable. There was no sign of meningeal irritation. Cardiovascular, respiratory and abdominal examinations were unremarkable.

The patient was admitted and relevant blood tests were ordered. Laboratory test results such as complete blood counts, serum electrolytes, thyroid profile, creatinine kinase, liver function test, lactate dehydrogenase (LDH), C-reactive protein, erythrocyte sedimentation rate (ESR) and serum B12 were normal. Anti-muscle-specific kinase (MuSK) antibodies and anti-acetylcholine receptor (ACHR) antibodies were also undetectable. Lumbar puncture was done for cerebrospinal fluid study which did not show any signs of active infection or albuminocytologic dissociation, however, IgM antibodies against Dengue antigen (NS 1) and Chikungunya virus (CHIKV) were detected, confirming the possibility of earlier Dengue-Chikungunya co-infection. Stool culture for Campylobacter jejuni was also negative. Magnetic resonance imaging (MRI) of the brain was ordered which did not show any pathological lesion. Subsequently, two days later, Nerve Conduction Studies (NCS) were performed on the upper and lower limb that showed normal sensory fiber conductions of median, ulnar, superficial/common peroneal and tibial nerve. However, there was decreased amplitude of the compound motor action potential (CAMPs), slowing of conduction velocity, prolonged distal latency and absent F-waves in the upper limbs (Table 1). On Electromyography (EMG), voluntary motor unit action potentials had normal morphology with diminished recruitment and normal spontaneous activity. These findings were consistent with the early stages of acute motor demyelinating and possible axonal neuropathy. This led to the diagnosis of the pharyngeal-cervical-brachial variant of GBS, occurring post-Dengue-Chikungunya co-infection. The diagnosis was further confirmed by the detection of antiganglioside antibodies (anti-GT1a antibodies).

**Table 1:** results of motor nerve conduction studies

Nerve	Stimulation	Recording	DL (msec)	Amplitude (mV)	Velocity (m/sec)	F-wave (msec)
Median	Wrist	APB	18.2	1.9	33.8	NR
	Elbow			1.5	32.2	
Ulnar	Wrist	ADM	19.1	2.0	32.1	NR
	Elbow			1.9	34.3	
Common peroneal	Ankle	EDB	3.3	6.2	61.2	R
	Fibular head			6.4	60.8	
Tibial	Ankle	AH	3.5	6.5	60.5	R
	Knee			6.4	61.1	

APB: abductor pollicis brevis, ADM: abductor digiti minimi, EDB: extensor digitorum brevis, AH: abductor hallucis, DL: distal latency (onset latency), NR: not recorded, R: recorded

From the 2^nd^ hospital day, the patient was started on the treatment of intravenous immunoglobulin (IVIG) with a dose of 0.4g/kg/day for a total of five days. Her symptoms partially improved after the five doses of IVIG. She did not experience any respiratory distress during her hospital stay and her vitals and oxygen saturation also remained stable. By the 11^th^ hospital day, the dysphagia was completely resolved and the patient was satisfied by this outcome. Muscle strength of the arms and neck were also assessed every day, which gradually improved to 3/5 by 16^th^ hospital day. Her physical rehabilitation was initiated and she was discharged after 15 days. The patient continued regular physiotherapy for the next four months. The MRC grade in both upper limbs and neck was 5/5 at her four-month of follow-up.

## Discussion

Guillain-Barré syndrome (GBS) is an acute monophasic polyneuropathy that can manifest in various ways, ranging from weakness in ambulation to complete paralysis of extremities, respiratory muscles necessitating ventilatory support, facial and bulbar muscles causing ophthalmoplegia, facial weakness or dysphagia, sensory deficits or dysautonomia. This shows that GBS is rather a heterogeneous syndrome with various variants [9,10]. Amongst those variants, pharyngeal-cervical-brachial (PCB), variant with the localized form of axonal loss- is an important and somewhat tricky variant to diagnose- given its overlapping features with myasthenia gravis, botulism, and brainstem stroke. Pharyngeal-cervical-brachial is usually diagnosed under Ropper diagnostic criteria, which the following postulates [9]: diagnostic criteria for the pharyngeal-cervical-brachial (PCB) variant of Guillain-Barré syndrome (GBS). Features required for diagnosis: relatively symmetric oropharyngeal weakness and neck weakness and arm weakness and arm areflexia/hyporeflexia; absence of ataxia and disturbed consciousness and prominent leg weakness; monophasic illness pattern and interval between onset and nadir of oropharyngeal or arm weakness between 12 h and 28 days and subsequent clinical plateau; absence of identified alternative diagnosis. Features strongly supportive of the diagnosis: antecedent infectious symptoms; cerebrospinal fluid albuminocytological dissociation; neurophysiological evidence of neuropathy; presence of IgG anti-GT1a or anti-GQ1b antibodies. The clinical severity of each component may vary from partial to complete.

Even though the absence of sensory symptoms and lack of lower extremity weakness is a part of the criteria, it should be noted that it might not always be the case. Upper extremity sensory loss is permitted, and so is the lower extremity weakness, but it shouldn't be the most predominant feature. Of note, apart from “pure PCB,” which follows the diagnostic criteria aforementioned, PCB can have a mixed presentation with frequent bulbar involvement- as it frequently overlaps Fischer syndrome and Bickerstaff encephalitis- two of the GBS variants [2]. Treatment usually involves disease-modifying therapies involving intravenous immunoglobin (IVIG) or plasma exchange with careful cardiorespiratory monitoring [11]. About two-thirds of GBS cases are post-infectious, with the viral illness being one of the most familiar triggers [12]. GBS has been noted after arboviral infections such as Zika virus (ZIKV), Dengue virus, and Chikungunya virus (CHIKV) - with cases surging during epidemic [13]. In part, GBS could be attributed to two mechanisms, either neuro-invasion given suspected neurotropism or an aberrant immune response to the peripheral nervous system leading to acute demyelination in the recovery period. Cross-reactivity has been noted in patients with ZIKV and dengue co-infection. The hypothesis of increased viral load or hyperimmune activation has been linked to GBS apart from molecular mimicry [14]. Immunoglobulin M antibodies to both dengue and CHIKV have been noted in cerebrospinal fluid, which could also explain neuro-invasion as one other mechanism. Even though there have been co-infections with dengue and CHIKV, no reports have been noted in literature regarding PCB variant of GBS. Also of note, GBS cases in arboviral illnesses have mostly been acute inflammatory demyelinating polyneuropathy, mono-infectious cases reported with PCB are rare [6,7,14,15].

**Informed consent:** informed consent regarding case publication was taken from the patient.

## Conclusion

Although GBS is a common neurological disorder, PCB is an unfamiliar variant for many clinicians. Diagnosis can be made by a thorough history, clinical examination and investigations that can rule out other potential causes of cervicobrachial and oropharyngeal weakness. Furthermore, it is important to remember that it can occur as a complication of mono- and co-arboviral infections, emphasizing the need for careful monitoring and follow-ups to prevent any debilitating neurological complications.
